# The clinical efficacy of laminectomy fusion fixation and posterior single open-door laminoplasty in the treatment of multilevel cervical ossification of the posterior longitudinal ligament (OPLL): a retrospective study

**DOI:** 10.1186/s12893-023-02289-9

**Published:** 2023-12-13

**Authors:** Qian Zhang, Rudan Guo, Sanhua Fang, Shunyi Tong, Yuan Fan, Jun Wang

**Affiliations:** Department of Orthopedics, Lanxi People’s Hospital, No.1359 Xishan Road, Lanxi City, Jinhua City, 321000 Zhejiang Province China

**Keywords:** OPLL, Multilevel, Laminectomy, Laminoplasty, Efficacy comparison

## Abstract

**Background:**

To compared the clinical efficacy of two surgical methods, posterior laminectomy fusion fixation, and posterior single open-door laminoplasty, in treating multilevel cervical ossification of the posterior longitudinal ligament (OPLL).

**Methods:**

The study retrospectively included 102 patients treated between December 2016 and December 2020. The patients were included into an observation group (56 cases) treated with total laminectomy and lateral screw fixation, and a control group (46 cases) treated with single open-door laminoplasty.

**Results:**

After 24 months, both groups showed significant improvement in Japanese Orthopaedic Association (JOA) scores and Visual Analogue Scale (VAS) scores, indicating better clinical symptoms and functional recovery. There was no significant difference in preoperative JOA and VAS scores between the two groups (*P* > 0.05). At 24 months after surgery, there was no significant difference in JOA and VAS scores between the two groups (*P* > 0.05). However, the observation group had a significantly higher cervical curvature index (CCI) and lower range of motion (ROM) of the cervical spine compared to the control group (*P* < 0.05). The CCI in control group was lower than before surgery, while the CCI in observation group was higher than before surgery, and CCI in the control group was considerably lower than that in the observation group (*P* < 0.05). The complication rate was lower in the control group, with fewer cases of axial symptoms, fifth cervical nerve root palsy, and overall complications. The overall complication rate was 25.0% (14/56) in the observation group and 10.8% (5/46) in the control group (*P* < 0.05).

**Conclusions:**

Both posterior laminectomy fusion fixation and posterior single open-door laminoplasty yield positive outcomes in improving clinical neurological function, cervical curvature, range of motion of the cervical spine, and cervical sagittal balance. Although open-door laminoplasty is less effective than total laminectomy in maintaining CCI and sagittal balance, it excels in preserving cervical range of motion, less surgical trauma and complications. Thus, open-door laminoplasty may be a suitable first-choice treatment for multi-segmental cervical OPLL, especially for patients with lordotic cervical spine physiological curvature.

## Background

The posterior longitudinal ligament is a rigid and narrow band located in the spinal canal behind the vertebral body of the spine. Its main function is to prevent excessive forward flexion of the spine and posterior prolapse of the intervertebral disc. Ossification of the posterior longitudinal ligament (OPLL) refers to the pathological phenomenon of abnormal hyperplasia, hypertrophy, and formation of bone structures in the posterior longitudinal ligament tissue [1]. The disease known as OPLL is brought on by pathological changes (heterotopic ossification) in the cervical spine’s (CS) posterior longitudinal ligament. OPLL often starts with no or mild symptoms, such as mild pain, tingling, and/or numbness in the hands [2]. It can also lead to dysesthesia, an uncomfortable sensation experienced upon touch [2]. As the condition worsens, symptoms gradually intensify. Symptoms of moderate to severe OPLL include difficulty walking and with bowel and bladder control (symptoms of myelopathy) [3]. The disease is a relatively common disease causing myelopathy in East Asian people at present [4, 5]. The incidence rate of this disease is 1.9% ~ 4.3%, accounting for about 0.54% ~ 0.88% of cervical spondylosis patients in China [5]. Literature has reported that the disease has significant regional distribution and racial differences, which may be related to genetic factors, metabolic factors, and dietary habits [4–6].

Patients with neurologic symptoms due to multilevel cervical OPLL usually require surgical intervention rather than conservative therapy. At present, surgical treatment mainly includes anterior, posterior, or combined approach [7]. The compression of spinal cord comes from the anterior and anterior surgery can remove the lesion directly and completely, interrupting the pathological progression. So some scholars believe that anterior surgery is the most effective and ideal approach, especially anterior cervical corpectomy, decompression, and fusion (ACCF) [8]. However, some authors have reported that both anterior and posterior approaches can lead to significant improvements in clinical outcomes [9]. Posterior surgeries like laminectomy and laminoplasty are commonly used to treat multiple-level cervical OPLL.

Multilevel cervical OPLL can be treated relatively safely and effectively with indirect posterior decompression. The principles are to remove or move vertebral lamina backward, expand vertebral canal, decompress spinal cord and avoid progression of ossification, thereby alleviating clinical symptoms and achieving decompression effect [10]. Common surgical methods include laminectomy and laminoplasty as well as laminectomy and fusion. Total laminectomy is gradually being abandoned due to its destruction of stable structures behind vertebral body during surgery which may lead to cervical kyphosis after surgery causing compression and stimulation of spinal canal tissue leading to symptom recurrence. At present scholars generally believe that laminoplasty can achieve good clinical efficacy while preserving posterior stability structure. At present, scholars generally recognize that both laminectomy fusion and laminoplasty can improve symptoms in patients with multi-level cervical OPLL. However, each surgical method has its own limitations and complications [11]. Laminectomy fusion is generally considered safe, but as with any surgery, it carries some risks. Possible complications of laminectomy fusion include bleeding, infection, blood clots, nerve injury, and spinal fluid leak [12].

There is currently a lack of effective statistical studies to analyze which of the two surgical methods is superior. This study focuses on the clinical efficacy of posterior cervical laminectomy fusion and laminoplasty in the treatment of multi-level cervical OPLL.

## Materials and methods

### General information

This retrospective study was approved by the Ethics Committee of our hospital. Written informed consents were obtained from all participants. This study included 102 patients with multi-level cervical OPLL who underwent posterior laminectomy fusion or laminoplasty in our department from December 2016 to December 2020. The curvature of the CS, the level of posterior longitudinal ligament ossification, and the related spinal cord compression were all assessed using preoperative anteroposterior and lateral X-ray films, CT, and magnetic resonance imaging (MRI) of the CS. The following are the precise inclusion and exclusion standards: inclusion standards: (1) The patients fulfilled the OPLL diagnostic requirements. (2) Patients had OPLL with progressive neurological deterioration. (3) Patients had three or more segments of cervical ossification. (4) The K-line (the line connecting the midpoint of the spinal canal of the second and seventh cervical vertebrae on the lateral cervical X-ray film) was positive. Ossification that crossed the K-line was defined as K-line negative, while ossification that did not cross the K-line was defined as K-line positive. (5) Clinical manifestations were medullary symptoms caused by OPLL compression of the spinal cord, and affected segments were consistent with clinical symptoms. (6) Conservative therapy was ineffective for more than three months. ***Exclusion criteria***: (1) Patients had only one or two segments of cervical OPLL. (2) Patients had obvious cervical kyphosis and were unsuitable for posterior approach. (3) Patients had ossification of the ligamentum flavum and OPLL in other parts of the spine. (4) Patients had a history of corresponding segment surgery or combined spinal deformity, tumor, infection, or thoracic OPLL. (5) Patients had incomplete postoperative follow-up data or follow-up time less than two years.

### Surgical methods

The patients in the laminectomy and fusion group were all given general anesthesia and placed in a prone position. Fluoroscopy was used to confirm that the patient’s CS was properly extended upward. A straight posterior incision was made at any length along with C3-7 spinous process, and paraspinal muscles were stripped bilaterally to expose bilateral laminae and facet joints. The cervical semispinous muscle attached to the second cervical spinous process and posterior ligament complex were retained. The C3-7 laminaes were completely cut off and removed bilaterally. The mode of internal fixation was 1 mm inward to the middle point of lateral mass, with a horizontal angle of 25° to lateral side and a cephalic tilt of 30–40° to open hole. Appropriate lateral mass screws were inserted at corresponding segments, and longitudinal connecting rods with appropriate pre-bending (pre-bending to physiological curvature of CS) were installed and fixed. Ultrasound osteotome was used to remove vertebral lamina for decompression, and bone graft fusion was performed at bilateral facet joints of C3-7 with bone cortex removed.

The laminoplasty group used side with severe spinal cord compression as opening side while preserving integrity of intervertebral disc, paraspinal muscles, and posterior ligament complex. A grinding drill was used to separate bone grooves and portal axis at connection between laminae and lateral mass, and opened laminae were supported with appropriately sized miniature titanium plates and screws. Both groups achieved full hemostasis. A drainage tube was inserted, and incision was sutured layer by layer.

### Postoperative management

After surgery, changes in patient’s vital signs were observed, hormone and antibiotic treatment were provided and maintained for 3–5 days. Neurological damage and incision hematoma were managed in a timely manner. According to color and quantity of patient’s wound drainage fluid, drainage tubes were removed in a timely manner. If cerebrospinal fluid leakage occurred intraoperatively, patient should be bedridden with head and feet in low position, incision should be appropriately pressurized, amount of cerebrospinal fluid leakage should be observed to prevent incision infection. Patients were protected with neck braces for 2–3 weeks, and slight CS activities such as nodding and turning head were performed as soon as possible.

These patients were followed up for at least 24 months.

### Observation indicators

#### Surgical complications

The patients were followed up for at least 24 months to observe incision infection, cerebrospinal fluid leakage, axial symptoms, fifth cervical nerve root palsy, and other complications.

#### Functional recovery evaluation

The neurological function of patients was assessed using the Japanese Orthopaedic Association (JOA) score before and 24 months after surgery. A higher JOA score indicates better neurological function [13].

#### Assessment of pain

The Visual Analogue Scale (VAS) was employed to assess axial pain in the neck and back before surgery and 24 months post-surgery. Pain intensity was represented by a scale ranging from 0 to 10, where 0 signified no pain, 1–3 indicated mild pain, 4–6 represented moderate pain, and 7–10 denoted severe pain. Patients selected a number on the scale corresponding to their level of pain [14].

#### Imaging assessment: cervical curvature index (CCI)

**Fig. 1:**
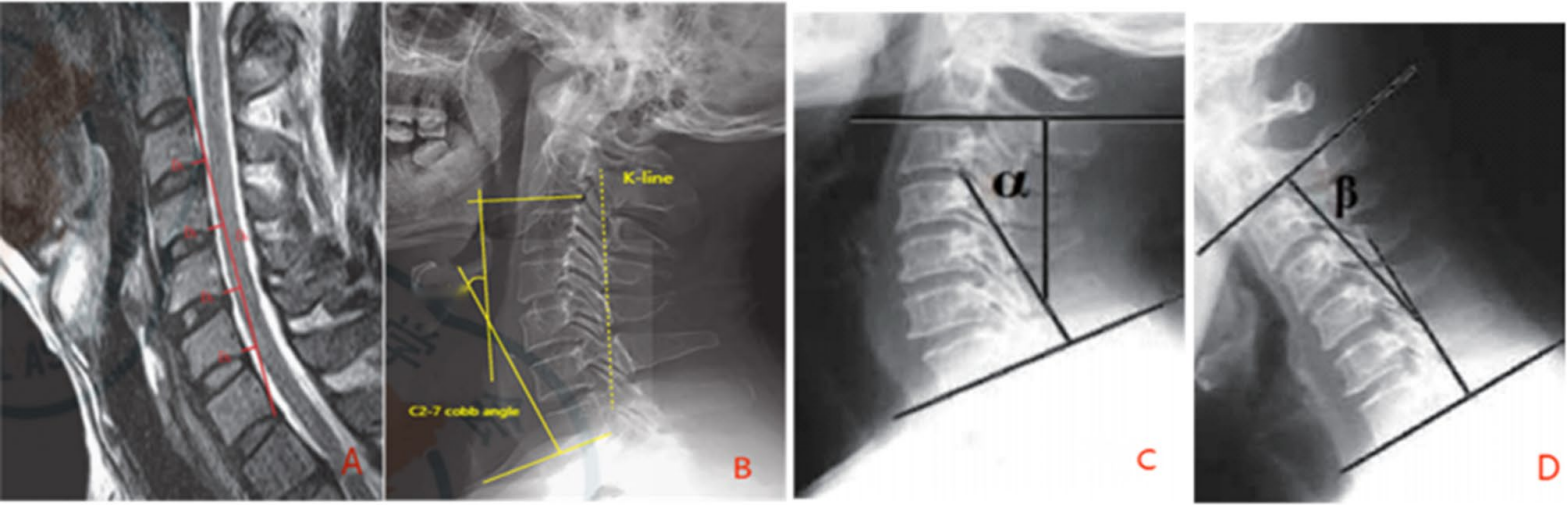
The cervical ROM mainly by taking dynamic X-ray film of cervical spine and measuring C2-7 Cobb Angle in flexion and hyperextension respectively. **A**. illustrates the schematic representation of CCI. The extent of postoperative CCI loss directly correlates with the quality of cervical curvature preservation; **B**. the diagram showcases the Cervical Sagittal Balance Index (C2-7 SVA) and the K-line. The deterioration in C2-7 SVA, calculated as the difference between postoperative and preoperative values, reflects a decline in sagittal balance within the cervical spine; Moving on to Pictures **C** and **D**, these diagrams depict the Range of Motion (ROM). The flexion angle, denoted as β, takes a negative value if the cervical spine exhibits a reverse curvature. Conversely, the hyperextension angle, labeled as α, contributes to the overall ROM, which can be calculated as ROM = α + β. The angle of loss is determined by the difference between preoperative and postoperative ROM values

A line from the posterior inferior margin of the second cervical vertebra to the posterior superior margin of the seventh cervical vertebra was marked as D0. The vertical distances D1, D2, D3, D4 from the posterior inferior margin of the third cervical vertebra to the posterior inferior margin of the sixth cervical vertebra to this line were measured. CCI = (D1 + D2 + D3 + D4) / D0. Loss of CCI = preoperative CCI - postoperative CCI; the smaller the loss of postoperative CCI, the better the preservation of CC [15]. ***Cervical Sagittal Balance Index (C2-7 SVA)***: This index reflects the sagittal balance of the CS and degree of CS anteversion. To measure it, take the vertical distance from posterior-superior edge of C7 vertebra to vertical line through center of second cervical vertebra on lateral X-ray of CS. The larger C2-7 SVA value, worse sagittal balance of cervical spine (SBCS). Increase in C2-7 SVA = postoperative C2-7 SVA - preoperative C2-7 SVA; greater increase in C2-7 SVA indicates worse SBCS [16]. ***ROM (Range of Motion***, Fig. 1***)***: The cervical ROM was mainly measured by taking dynamic X-ray film of CS and measuring C2-7 Cobb Angle in flexion and hyperextension respectively [17]. Angle of flexion was β; if CS was in reverse, β was negative. Angle of hyperextension was α; ROM = α + β. Preoperative ROM - postoperative ROM = Angle of loss.

### Statistical analysis

Software called SPSS 22.0 was used for the statistical analysis. Measurement data were shown as mean ± standard deviation (mean ± SD) and were subjected to t-test analysis, whilst count data were displayed as percentages and were subjected to 2 tests. The correlation between age, ROM, CCI, C2-7 SVA and neurological function recovery was analyzed by multivariate logistic regression analysis. Statistical significance was defined as a p-value of less than 0.05.

## Results

**Fig. 2:**
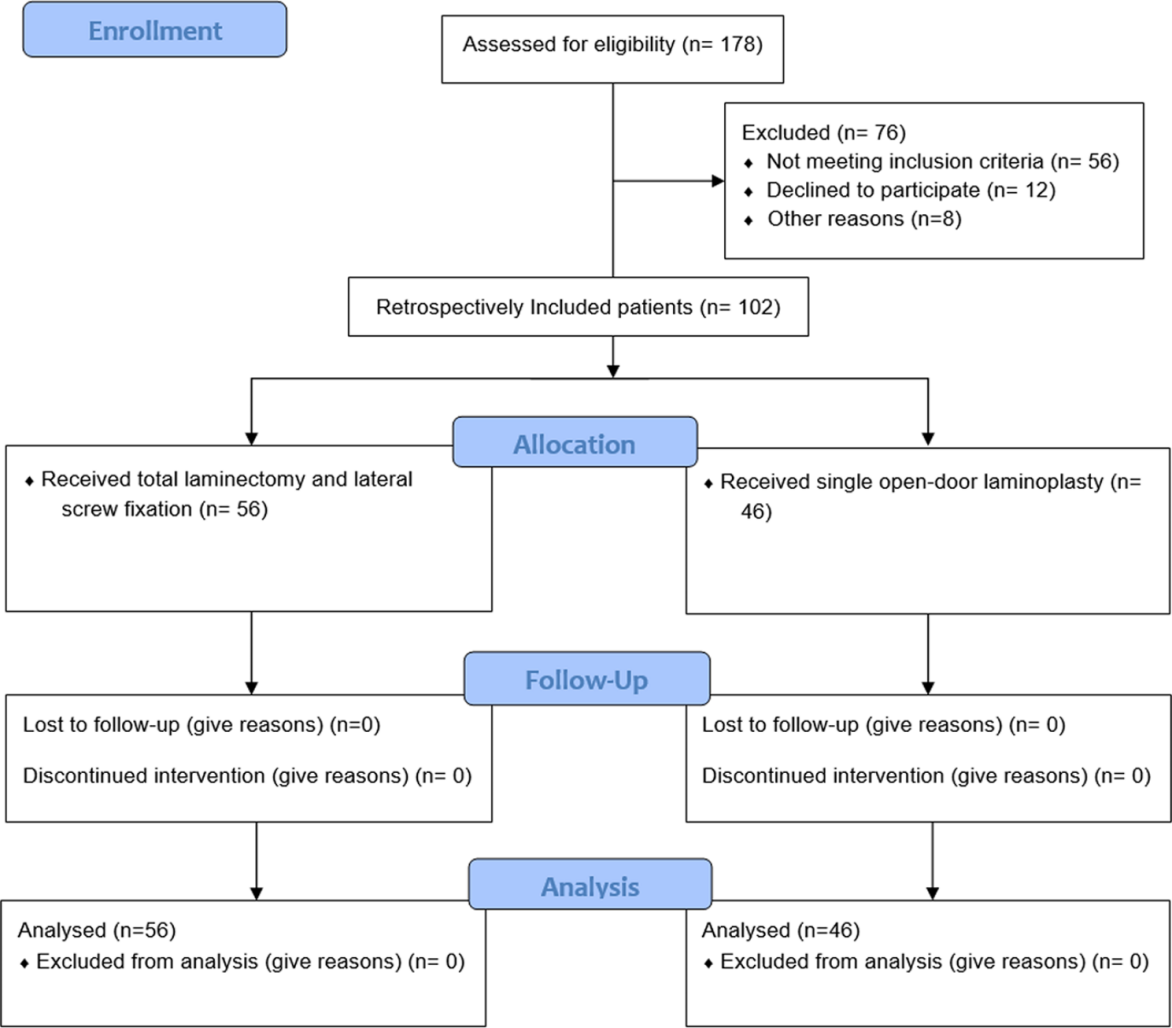
Flow chart of included patients

A total of 102 patients were included, as shown in Fig. 2. The age range of the patients was 53 to 80 years, and the condition lasted an average of 3.2 ± 1.3 years, ranging from 3 months to 8 years. The laminectomy and fusion group had 56 cases, with 31 men and 25 women, ranging in age from 49 to 66 (57.75 ± 4.72) years. This group had four cases of diabetes and five cases of hypertension. The laminoplasty group included 46 cases, with 24 males and 22 females, aged between 43 and 70 (58.77 ± 6.45) years. There were two cases of diabetes and three cases of hypertension in this group. No significant differences were noticed in gender, age, diabetes, or hypertension between the two groups (*P* > 0.05).

### General situations during operation

Table 1 compares the perioperative indexes of the laminectomy group and the laminoplasty group. The incision length was slightly longer in the laminectomy group (11.37 ± 1.35) compared to the laminoplasty group (10.49 ± 1.08), but the difference was statistically insignificant (*P* > 0.05). The operation time was significantly longer in the laminectomy group (224.86 ± 43.7) compared to the laminoplasty group (164.86 ± 36.77), with a statistically substantial difference (*P* < 0.05). Blood loss was also significantly higher in the laminectomy group (307.88 ± 62.18) compared to the laminoplasty group (201.45 ± 54.16), with a statistically substantial difference (*P* < 0.05). Postoperative drainage was significantly higher in the laminectomy group (207.42 ± 21.39) compared to the laminoplasty group (103.42 ± 10.28), with a statistically substantial difference (*P* < 0.05).

**Table 1:** The comparison of the perioperative indexes of the two groups (mean ± SD)

Index	Lanminectomy group	Laminoplasty group	t value	*P* value
Incision length	11.37 ± 1.35	10.49 ± 1.08	3.473	*P* > 0. 05
Operation time	224.86 ± 43.7	164.86 ± 36.77	5.050	*P* < 0. 05
Blood loss	307.88 ± 62.18	201.45 ± 54.16	6.627	*P* < 0. 05
Postoperative drainage	207.42 ± 21.39	103.42 ± 10.28	7.356	*P* < 0. 05

### The results of JOA and VAS scores of the two groups before and after operation

Table 2 compares the JOA and VAS scores between the laminectomy group and the laminoplasty group before and 24 months after surgery. The preoperative JOA scores were similar between the two groups, with 8.11 ± 1.06 in the laminectomy group and 8.03 ± 0.89 in the laminoplasty group, and the difference was statistically insignificant (*P* > 0.05). At the last follow-up, the JOA scores had improved in both groups, with 12.08 ± 1.36 in the laminectomy group and 12.37 ± 1.43 in the laminoplasty group, but the difference between the two groups was statistically insignificant (*P* > 0.05). The improvement in JOA scores from pre-operation to last follow-up was statistically significant in both groups (*P* < 0.05).

**Table 2:** Comparison of JOA and VAS scores between two groups of patients before and 24 months after surgery (mean ± SD)

Index	Point-in-time	Lanminectomy group	Laminoplasty group	T value	*P* value
JOA score	Pre-operation	8.11 ± 1.06	8.03 ± 0.89	1.732	*P* > 0. 05
	Last follow-up	12.08 ± 1.36	12.37 ± 1.43	1.845	*P* > 0. 05
	T value	8.590	8.783		
	*P* value	*P* < 0. 05	*P* < 0. 05		
VAS score	Pre-operation	6.75 ± 1.23	7.03 ± 1.45	2.375	*P* > 0. 05
	Last follow-up	2.33 ± 0.63	2.07 ± 0.52	2.689	*P* > 0. 05
	T value	23.179	29.875		
	*P* value	*P* < 0. 05	*P* < 0. 05		

Preoperative VAS scores were comparable in both groups, with laminectomy values 6.75 ± 1.23 and laminoplasty ratings 7.03 ± 1.45; the difference was statistically insignificant (*P* > 0.05). The VAS scores had increased at the most recent follow-up, with the laminectomy group having 2.33 ± 0.63 and the laminoplasty group having 2.07 ± 0.52, respectively. However, the difference between the two groups was statistically insignificant (*P* > 0.05). In both groups, the change in VAS scores from the baseline to the final follow-up was statistically significant (*P* < 0.05).

### The results of CCI, C2-7 SVA, and ROM before and after operation

Table 3 compares the CCI, C2-7 SVA, and ROM between the laminectomy group and the laminoplasty group before and after surgery. The preoperative CCI was similar between the two groups, with 12.73 ± 1.79 in the laminectomy group and 12.65 ± 1.50 in the laminoplasty group, and the difference was statistically insignificant (*P* > 0.05). The CCI had increased in observation group and decreased in control group at the last follow-up, with the laminectomy group having an increased CCI of 17.36 ± 1.78 and the laminoplasty group having a decreased CCI of 10.72 ± 1.63. This difference between the two groups was statistically significant (*P* < 0.05). The difference in CCI between the two groups from pre-operation to last follow-up was statistically inconsequential (*P* > 0.05), whereas the difference between the two groups from pre-operation to final follow-up was statistically significant (*P* < 0.05).

**Table 3:** Comparison of CCI, C2 -7 SVA, ROM between the two groups before and after operation (mean ± SD)

Index	Point-in-time	Lanminectomy group	Laminoplasty group	T value	*P* value
CCI	Pre-operation	12.73 ± 1.79	12.65 ± 1.50	0.947	*P* > 0.05
	Last follow-up	17.36 ± 1.78	10.72 ± 1.63	3.679	*P* < 0. 05
CCI change		5.38 ± 1.25	− (1.93 ± 1.27)	1.179	P<0. 05
	T value	10.637	3.245		
	*P* value	*P* < 0. 05	*P* < 0. 05		
C2-7 SVA	Pre-operation	18.13 ± 4.20	21.94 ± 3.18	3.274	*P* > 0. 05
	Last follow-up	25.00 ± 3.82	16.41 ± 2.78	16.285	*P* < 0. 05
C2-7 SVA change		7.81 ± 2.80	− (8.58 ± 2.91)	13.491	*P* < 0. 05
	T value	10.235	18.642		
	*P* value	*P* < 0. 05	*P* < 0. 05		
ROM	Pre-operation	26.58 ± 3.50	28.06 ± 3.79	2.798	*P* > 0. 05
	Last follow-up	13.36 ± 3.78	22.95 ± 2.71	36.251	*P* < 0. 05
ROM change		13.23 ± 4.35	5.10 ± 3.23	12.350	*P* < 0. 05
	T value	3.0725	16.329		
	*P* value	*P* < 0. 05	*P* < 0. 05		

With 18.13 ± 4.20 in the laminectomy group and 21.94 ± 3.18 in the laminoplasty group, the preoperative C2-7 SVA was equally comparable between the two groups and the difference was statistically insignificant (*P* > 0.05). The C2-7 SVA had increased in observation group and decrease in control group at the most recent follow-up, with the laminectomy group seeing an increase of 25.00 ± 3.82 and the laminoplasty group experiencing an decrease of 16.41 ± 2.78; the difference between the two groups was statistically significant (*P* < 0.05). Although the change in C2-7 SVA from pre-operation to last follow-up was statistically significant in both groups (*P* < 0.05), the increase in C2-7 SVA was statistically significant between the two groups (*P* < 0.05).

The preoperative ROM was similar between the two groups, with 26.58 ± 3.50 in the laminectomy group and 28.06 ± 3.79 in the laminoplasty group, and the difference was statistically insignificant (*P* > 0.05). At the last follow-up, ROM had decreased in both groups, with 13.36 ± 3.78 in the laminectomy group and 22.95 ± 2.71 in the laminoplasty group, and the difference between the two groups was statistically considerable (*P* < 0.05). The decrease in ROM from pre-operation to last follow-up was statistically significant in both groups (*P* < 0.05). Additionally, the loss of ROM from pre-operation to last follow-up showed a significant difference between the two groups (*P* < 0.05).

### Postoperative complications

After 24 months of follow-up, the laminectomy group had a total complication rate of 25.0% (14/56), with 2 cases (3.57%) of incision infection, 2 cases (3.57%) of cerebrospinal fluid leakage, 5 cases (8.9%) of axial symptoms, and 5 cases (8.9%) of fifth cervical nerve root numbness. The laminoplasty group had a total complication rate of 10.8% (5/46), with 1 case (8.3%) of incision infection, 1 case (8.3%) of cerebrospinal fluid leakage, 2 cases (2.8%) of axial symptoms, and 1 case (5.6%) of fifth cervical nerve root palsy.

Table 4 compares the postoperative complications between the laminectomy group and the laminoplasty group two years after surgery. The incidence of incision infection, cerebrospinal fluid leakage, axial symptoms, and fifth cervical nerve root numbness were all higher in the laminectomy group compared to the laminoplasty group, and the differences were statistically significant (*P* < 0.05). Overall, the laminectomy group experienced more postoperative problems (14 cases) than the laminoplasty group did (5 cases), and the difference was statistically significant (*P* < 0.05).

**Table 4:** Comparison of postoperative complications between the two groups two years after operation (cases)

Complications	Lanminectomy group	Laminoplasty group	χ2 value	*P* value
Incision infection	2	1	5.119	*P* < 0. 05
Cerebrospinal fluid leakage	2	1	5.119	*P* < 0. 05
Axial symptoms	5	2	6.829	*P* < 0. 05
Fifth cervical nerve root numbness	5	1	8.374	*P* < 0. 05
Total	14	5	9.347	< 0. 05

### Factors related to postoperative neurological function

The findings of a multivariate logistic regression analysis of patients with no appreciable improvement in neurological function are displayed in Table 5. The independent variables included in the analysis were age, ROM, CCI, and C2-7 SVA. The results show that ROM was a significant predictor of no significant improvement in neurological function, with a β value of 1.479, a standard error of 0.419, a Wald value of 4.926, a p-value of 0.019, an OR value of 7.493, and a 95% CI of 4.236–17.349. Age, CCI, and C2-7 SVA were not significant predictors of no significant improvement in neurological function.

**Table 5:** The results of Multivariate logistic regression analysis of patients with no significant improvement in neurological function

Independent variable	β	standard error	Wald value	*P* value	OR	95%CI
Age (y)	1.049	0.530	4.374	0.347	2.372	0.794–11.437
ROM	1.479	0.419	4.926	0.019	7.493	4.236–17.349
CCI	1.194	0.437	5.134	0.539	1.948	0.429–20.048
C2-7 SVA	1.278	0.544	4.348	0.098	5.988	0.972–14.349

## Discussion

Multiple surgical methods are available for treating multi-level cervical OPLL, such as anterior cervical corpectomy and fusion (ACCF), posterior laminectomy and fusion, posterior laminoplasty, combined surgery, or anterior controllable ante-displacement and fusion (ACAF) [18, 19]. ACAF involves moving the vertebral body and ossification forward as a whole, expanding the spinal canal’s volume, relieving spinal cord compression, and effectively avoiding the risks associated with traditional anterior resection of ossification. However, accurate long-term follow-up is still needed to confirm the occurrence of complications and assess long-term clinical efficacy [20]. At present, there is still controversy about the optimal surgical treatment for multi-level cervical OPLL.

Improved surgical techniques have made anterior resection the most effective method for OPLL, but it has risks for long-segment cases. Chen et al. suggested that anterior surgery requires interbody fusion of multiple segments, reducing CS motion, leading to adjacent segment degeneration, fixation failure, and bone graft nonfusion. For safer outcomes, posterior cervical laminectomy decompression, bone graft fusion, and internal fixation or laminoplasty are preferred. These methods offer long decompression segments, relatively simple operation, lower surgical risk, and good restoration of CC. Although posterior decompression cannot remove anterior compression factors, it achieves more extensive decompression indirectly, shifting the spinal cord backward. Long-term follow-up shows good CS stability. Factors like age, preoperative injury, and ossification of the ligamentum flavum can impact laminectomy efficacy. Additionally, cervical kyphosis and intervertebral instability can stimulate OPLL progression [21–23].

The surgical techniques laminectomy fusion and laminoplasty are performed to treat spinal stenosis, a condition in which the spinal canal narrows and compresses the spinal cord and nerves. By making greater space in the spinal canal, both operations aim to relieve pressure on the spinal cord and nerves. The lamina, which is the rear section of a vertebra that surrounds the spinal canal, is removed during laminectomy fusion. This expands the spinal canal and lowers spinal cord pressure. Following a laminectomy, the damaged vertebrae are bonded together with bone grafts, screws, and rods. This stabilizes the spine and keeps the fused vertebrae from moving [24].

Laminoplasty, on the other hand, involves repositioning the lamina instead of completely removing it. The lamina is cut on one side and partially cut on the other side, creating a hinge. The lamina is then lifted and held in place with small metal plates and screws, creating more space in the spinal canal [25]. Unlike laminectomy fusion, laminoplasty maintains a patient’s range of motion [25]. One study found that laminectomy fusion was associated with longer operative durations, higher complication rates, more blood loss during surgery, and decreased range of motion in the neck when compared to laminoplasty [26]. However, it is important to note that every patient is unique and the best surgical approach should be determined by a qualified medical professional based on individual circumstances.

In the posterior cervical laminectomy decompression and fusion group, on the basis of sufficient posterior decompression, the lateral mass system was used to control intervertebral movement of ossified segment while maintaining original curvature of CS to avoid late kyphosis. Japanese scholars Fujiyoshi et al. [27] used K-line to determine relationship between spinal canal encroachment rate, CC and choice of surgical methods and efficacy. They proposed that K-line negative patients underwent posterior cervical surgery because insufficient dorsal retreat of spinal cord resulted in poor recovery of spinal nerve function. Therefore, this study was performed on basis of positive K-lines. In this study, postoperative JOA scores in both groups were higher than before operation but there was no statistical difference between two groups before and after operation. This confirmed that these two posterior CS surgery methods can improve clinical neurological function in patients, reduce pain, and achieve satisfactory clinical results.

The preoperative physiological curvature of the CS plays an imperative role in the “bowstring effect” after posterior decompression of the spinal cord, which is the theoretical basis for the indirect decompression of the posterior surgery. However, the removal of the posterior ligament and muscle complex by the posterior surgery will lead to the loss of CC and cervical instability [28]. The CS imbalance may result in re-compression of the spinal cord and neurological degeneration. In the investigation of open-door laminoplasty and total laminectomy decompression in the treatment of multi-level cervical OPLL, Highsmigt et al. [29] and Yang et al. [30] found no evident kyphosis, and the CC was generally well preserved during the follow-ups. Total laminectomy decompression can effectively reduce posterior ossification pressures on the spinal cord while maintaining CS curvature. According to Duan et al. [31], the screw rod system can create a powerful three-dimensional biomechanical environment for the CS following total laminectomy, which is useful in restoring the CS’s physiological lordosis. In this study, the postoperative CCI of patients in both groups was lower than before surgery, and the CC was lost to varying degrees during the follow-up, which could be attributed to the surgical trauma that destroyed the stability of the CS and accelerated CS degeneration. However, CCI loss was drastically higher in the laminoplasty group than in the laminectomy group, which was thought to be due to laminoplasty lifting the lamina along the bone groove on the portal axis side, destroying the integrity of the posterior column of the CS, and accelerating CS instability. Total laminectomy can restore CS stability with lateral mass screws and restore CS physiological curvature with pre-bending titanium rods, decreasing CC loss.The C2-7 SVA of the laminectomy group was considerably higher than that of the control group, while the control group’s CCI and C2-7 SVA were better than those of the observation group. C2-7 SVA is a factor that represents the CS’s sagittal equilibrium. The loss of CC is caused by open-door laminoplasty, and the destruction of the posterior ligament aggravates the change in sagittal balance of the CS and increases the tendency of the CS to lean forward, resulting in the destruction of the sagittal balance of the CS. Total laminectomy and fusion, on the other hand, can retain the CC well, making the cervical sagittal balance less impaired. Tang et al. [32] found that the recovery of neurological function was related to C2-7 SVA, and the deterioration of neurological function occurred when C2-7 SVA exceeded 40 mm. In this study, the postoperative C2-7 SVA increased in both groups, but it did not cause neurological deterioration, which may be related to the C2-7 SVA not exceeding the risk value of “40 mm”, but the long-term efficacy needs further observation.

Compared to total laminectomy decompression, open-door laminoplasty is favored for its preservation of range of motion (ROM) and simplicity. It avoids excessive bone and muscle ligament resection behind the neck, reducing the risk of adjacent segment degeneration. Open-door laminoplasty has become a common procedure for multi-level OPLL. Preserving the bone and ligament structures in the posterior CS ensures better stability for maintaining the spinal canal formation, enabling early ambulation post-surgery. Maintaining cervical ROM is essential for normal physiological function and improved quality of life. Yuan et al. [33] compared different surgical interventions on ROM and found that open-door laminoplasty provided greater ROM than total laminectomy decompression and fusion. The loss of ROM after posterior cervical surgery primarily depends on the “spinous process-ligament-muscle complex,” with laminoplasty causing less loss than laminectomy. Although laminectomy offers immediate stability and curvature recovery, it significantly reduces CS ROM due to bone fusion. In contrast, laminoplasty preserves ROM, leading to less neck stiffness and minimal impact on quality of life and neck function. The use of a long segment titanium rod for fixation and bone graft fusion in total laminectomy and decompression patients offers stability but sacrifices most CS ROM [34].

Axial symptoms are mainly pain from the neck to the surrounding clavicle area or shoulder, which is a common complication of posterior cervical surgery. The cause is not clear, and may be related to the damage of soft tissue such as the posterior ligament and muscle of the neck and the decrease of the ROM of the CS during the operation [35].In this study, there are still a few patients with axial symptoms after surgery, which is considered to be related to the inevitable destruction of the posterior ligament complex during the operation and the loss of postoperative CS movement. Therefore, careful operation and strict entry along the white line during the operation to reduce the dissection of normal tissue structures are very important to reduce postoperative axial symptoms. Du et al. [36] reported that patients were encouraged to perform neck functional exercise early after surgery, and shortening the time of wearing neck brace could also reduce the occurrence of postoperative axial symptoms. Yang et al. [37] showed that the incidence and degree of axial symptoms after laminoplasty were lower than those after total laminectomy, decompression, fixation and fusion. However, this study showed that the incidence of axial symptoms after laminoplasty was slightly higher than that after laminectomy, decompression, fixation and fusion (2.3% vs. 1.2%). This conclusion may be due to the multi-center source of case data, different operators and case collectors.

An average of 8.3% (range: 3.2-28.6%) of patients experience C5 nerve root palsy as a common complication after posterior cervical surgery [38]. It is mainly manifested as decreased muscle strength of deltoid and biceps brachii, accompanied by decreased or absent sensation in the shoulder and lateral arm and weakened or absent biceps tendon reflex. The majority of complications (92%) occurred unilaterally within 1 day to 1 week after the operation, with a few (8%) occurring bilaterally within 2 to 4 weeks post-operation. Nerve root tethering effect is considered to be the main pathological mechanism of C5 nerve root palsy [39]. An et al. [40] also believed that the occurrence of OPLL was also related to the poor preoperative cervical physiological curvature of OPLL patients. During posterior laminectomy decompression, bone grafting, fusion and internal fixation, the cervical lordosis should be reconstructed to make the spinal cord drift backward and reduce the compression of the spinal cord, but at the same time, the traction of the nerve root can not be avoid. Liu et al. [41] proposed that C5 nerve root palsy might be linked to ossification and ligament adhesion around the nerve root outlet in OPLL patients. The incidence of C5 nerve root palsy after posterior cervical laminectomy with internal fixation fusion was comparable to cervical laminoplasty (2.4% vs. 3.0%). The observed group had a significantly lower occurrence of fifth cervical nerve root palsy, possibly due to nerve root traction caused by the spinal cord’s backward drift. Total laminectomy decompression enlarged the spinal canal and increased the spinal cord’s backward drift, leading to a higher occurrence of C5 nerve root palsy. Due to the significant paraspinal muscle dissection necessary for total laminectomy decompression with lateral mass screws, which led to high postoperative complication rates, the observation group experienced less issues than the control group.

After C5 nerve root palsy, bed rest, neck immobilization, hormone, dehydrating agent and neurotrophic agent can be used to recover quickly. For patients with severe muscle paralysis, the recovery time is longer. Generally, C5 nerve root palsy was treated by cervical traction to reduce CC and reoperation to remove part of the facet joint to open the intervertebral foramen after 6 months to 1 year. A total of 6 patients in this study were mainly treated with muscle function rehabilitation training after operation, and severe cases were treated with hyperbaric oxygen adjuvant therapy, which shortened the rehabilitation time. At the 1-year follow-up after operation, their muscle strength recovered to grade 3–4, and they could take care of themselves, and the rehabilitation effect was ideal.

However, the study has limitations in sample size, follow-up period, and lack of detailed comparative analysis. In addition, The K line (-) patients have been excluded in this study, which may be a limited aspect, too. Moreover, there might be a selection bias for choosing whether laminectomy and fusion or laminoplasty. More research is needed to strengthen the findings and determine the most suitable surgical approach for OPLL patients.

## Conclusion

Both posterior laminectomy fusion fixation and posterior single open-door laminoplasty yield positive outcomes in improving clinical neurological function, cervical curvature, range of motion of the cervical spine, and cervical sagittal balance. Although open-door laminoplasty is less effective than total laminectomy in maintaining CCI and sagittal balance, it excels in preserving cervical range of motion, less surgical trauma and complications. Thus, open-door laminoplasty may be a suitable first-choice treatment for multi-segmental cervical OPLL, especially for patients with lordotic cervical spine physiological curvature.

## Data Availability

All data generated or analysed during this study are included in this published article.

## Abbreviations

OPLL: Ossification of the posterior longitudinal ligament
JOA: Japanese Orthopaedic Association
VAS: Visual Analogue Scale
ROM: Range of motion
CCI: Ervical curvature index
CS: Cervical spine’s
ACCF: Anterior cervical corpectomy, decompression, and fusion
CC: Cervical curvature
LF: Laminectomy and fusion
MRI: Magnetic resonance imaging
